# Protocol of a cluster randomised trial of BodyKind: a school-based body image programme for adolescents

**DOI:** 10.1186/s12889-023-17002-x

**Published:** 2023-11-14

**Authors:** Ciara Mahon, Denise Hamburger, Jennifer B. Webb, Zali Yager, Emma Howard, Ailbhe Booth, Amanda Fitzgerald

**Affiliations:** 1 School of Psychology, University College Dublin (UCD), Newman Building, Belfield, Dublin 4, Ireland; 2 Be Real USA, Chicago, NFP USA; 3 Department of Psychological Science, Health Psychology Ph.D. Program, University of North Carolina at Charlotte (UNC Charlotte), Charlotte, USA; 4 Embrace Collective NFP, Adelaide, Australia; 5 School of Computer Science and Statistics, Trinity College Dublin, Dublin, Ireland; 6 Jigsaw, The National Centre for Youth Mental Health, Dublin, Ireland

**Keywords:** Body image, Adolescent, Intervention, Protocol, School-based, Psychological wellbeing, Body dissatisfaction, Randomised control trial, Implementation evaluation

## Abstract

**Background:**

Poor body image is prevalent among adolescents and associated with several negative outcomes for their physical and psychological health. There is a pressing need to address this growing public health concern, yet there are few evidence-informed universal programmes for older adolescents that address contemporary body image concerns (i.e., social media). BodyKind is a four lesson, school-based, teacher led, universal body image programme that incorporates empirically supported principles of cognitive dissonance, self-compassion, compassion for others and social activism, to support positive body image development. Building on previous pilot trials in the USA, this paper outlines the protocol for a cluster randomised control trial (cRCT) and implementation evaluation of the BodyKind programme which was culturally adapted for the Irish cultural context.

**Methods:**

We aim to recruit 600 students aged 15-17 years in Transition Year (4^th^ year) across 26 second-level schools in Ireland. Using minimisation, schools will be randomly assigned to receive BodyKind (intervention condition, *n=*300) or classes as usual (waitlist control, *n=*300). Teachers in intervention groups will receive training and deliver the programme to students over four weeks, at a rate of one lesson per week. Primary outcomes of body appreciation, body dissatisfaction and psychological wellbeing and secondary outcomes of self-compassion, compassion for others, body ideal internalisation, social justice motives and appearance-based social media use will be assessed at pre-, post- and 2 month follow up. Mediation and moderation analyses will be conducted to identify how and for whom the intervention works best. An implementation evaluation will assess the quality of programme implementation across schools and how this may influence intervention outcomes. Waitlist control schools will receive the programme after the 2-month follow up.

**Conclusion:**

This study will be the first to implement a cRCT and an implementation evaluation to assess the impact of this multicomponent school-based body image programme designed to support healthy body image development. If shown to be effective, BodyKind will have the potential to improve adolescent body image and wellbeing and inform efforts to implement sustainable and scalable programmes in schools.

**Trial registration:**

The trial was retrospectively registered on 10/10/2023 on ClinicalTrials.gov NCT06076993.

**Supplementary Information:**

The online version contains supplementary material available at 10.1186/s12889-023-17002-x.

## Background

Body image concerns are prevalent among adolescents, with an estimated 75% of young people reporting body image distress worldwide [1]. Body image concerns are associated with several negative outcomes for physical health [disordered eating/exercise [2, 3] and psychological wellbeing [low self-esteem, negative affect [3, 4] and are a primary modifiable risk and maintenance factor for eating disorders [5, 6]. Body image concerns and eating disorders have increased over the last decade [1, 7], particularly since the Covid-19 pandemic, with some studies reporting almost a doubling in the incidence of eating disorder related care in 2019 for adolescents compared with previous years [8–10]. Similarly in Ireland, less than half of adolescents are satisfied with their appearance [11] and a 66% increase in acute hospital admissions for eating disorders was observed among young female adolescents in Ireland between 2019-2020 [12]. Effective prevention is required to reduce the burden of disease and support adolescent psychological wellbeing [13]. Universal eating disorder prevention, which addresses all levels of risk, are often delivered in schools as they provide a cost-effective and inclusive way to access a wide range of adolescents within a sustained, learning environment [13, 14].

Traditionally, eating disorder prevention approaches have targeted risk factors for body image concerns [15], such as body ideal internalisation [i.e., cognitively endorsing body ideals as personal body standards [16] and appearance comparisons [i.e., comparing oneself on dimensions of appearance [17], which according to the Tripartite Model of Body Image [18], mediate the relationship between sociocultural appearance pressures (e.g., social media, peers, family) and the development of body image concerns. There is considerable evidence that such cognitive dissonance approaches [19], which involve publicly criticising unrealistic body ideals reduce the pursuit (internalisation) and comparison with these ideals, are effective in reducing adolescent body dissatisfaction in school-based trials [14, 15, 20, 21].

Recently, prevention approaches have acknowledged the importance of promoting positive body image, in addition to countering body dissatisfaction [22]. Positive body image, which is operationalised as body appreciation, is a unique, holistic construct which involves respecting, appreciating, nurturing and caring for one’s body and honouring natural body diversity [23], and is independently associated with benefits for physical and psychological health [e.g., greater adaptive coping, life-satisfaction, self-care behaviours [22–24]. Self-compassion represents a promising approach for supporting body appreciation and psychological wellbeing [25, 26]. Self-compassion is an emotional regulation strategy [27] that enables individuals to self-soothe by reframing self-critical thoughts and shame that are at the root of body dissatisfaction [28]. Self-compassion also helps individuals to appreciate alternative aspects of themselves (rather than overvaluing appearance) to promote positive body image [27, 29]. Self-compassion interventions are found to be effective in supporting adolescent psychological wellbeing [30, 31] and there is growing evidence that they show promise for improving body appreciation in adolescents [32–34].

Additionally, social justice perspectives [35], advocate that eating disorder prevention should move beyond the individual, to target the broader structural and social inequities inherent in diet culture that initiate and perpetuate appearance concerns in the first place [36]. Within the classroom context, where peers are present, empowering adolescents to challenge appearance biases (where people are treated differently based on how they look versus how they are as a person) and to engage in prosocial, compassionate behaviours towards others, such as challenging peer norms or reducing body talk/body shaming, could represent an approach to develop a supportive context for positive body image development [37–40]. However, further research on social justice motives and prosocial body image behaviours as pathways to building positive body image in adolescents are required [37].

While progress has been made in school-based body image intervention approaches, many gaps remain; firstly, most existing evidence-based programmes target early adolescents (12–13-year-olds), but there are fewer programmes that address body image concerns of older adolescents, despite the finding of the peak onset of eating disorders is between mid-late adolescence [41, 42]. Additionally, many existing programmes fail to address contemporary adolescent body image concerns, such as social media-related concerns [although there are exceptions [43, 44], or do not focus on the body image concerns of males and adolescents across the gender spectrum [15]. Also, many interventions are researcher-led, and there is a need for scalable and self-sustainable effective programmes that are teacher-led and that work towards building contexts to support positive body image development [36, 45].

Be Real’s BodyKind is a four-session, teacher-led, gender-inclusive school-based programme for adolescents aged 15-17 years that targets contemporary body image issues for adolescents (e.g., social media, gender inclusivity). This multicomponent intervention combines, for the first time, empirically supported principles of cognitive dissonance, self-compassion and social activism and is anticipated to enhance body image outcomes by facilitating various mechanisms of change to occur [outlined elsewhere in a Logic Model [46]. BodyKind was originally developed for high schools in the USA, with goals for broader, global implementation should the programme prove efficacious. Preliminary trials in the USA indicate the acceptability and feasibility of the BodyKind programme [46]. To facilitate the next phase in the development/evaluation of complex interventions, a rigorous cluster randomised trial cRCT evaluation across multiple sites is required [47].

To this end, BodyKind was culturally adapted to the Irish context via a series of codesign workshops with adolescents (*n=*12, 15-16 years) and interviews with teachers (*n=*6) and a clinician (*n=*1) to optimize intervention effects [48]. After receiving the BodyKind programme, students shared perceptions of programme acceptability and codesigned content to ensure its cultural relevance to the Irish context (e.g., examples/scenarios that are relevant for young people in Ireland) [49, 50]. Teacher feedback was used to facilitate implementation in schools (i.e., scheduling content delivery across 40–60-minute class durations, and to ensure content aligned with the Department of Education and Skills Wellbeing Policy Statement and Framework for Practice). The programme was considered highly acceptable by teachers and students, with 82% of students stating that they enjoyed the lessons and 73% would recommend the programme to a friend. A female clinician from Jigsaw, The National Centre for Youth Mental Health, was also consulted to ensure the programme aligned with Jigsaw’s ethos for supporting youth mental health in Ireland. Cultural adaptation protocols and details of changes made to the programme are outlined elsewhere.

The current paper describes the protocol for a cluster randomised control trial cRCT, which will be conducted in Irish schools with 4th year second-level students (aged 15-17 years) to evaluate the effectiveness of the culturally adapted BodyKind programme in improving body image and mental health outcomes among adolescents in Ireland. Schools will be randomly assigned to intervention or waitlist control conditions [14, 43]. Teachers in the intervention condition will complete 2.5 hours of training and will deliver the programme to students. Primary outcomes of body image and psychological wellbeing, and secondary outcomes of broader body image risk and protective factors will be assessed at three time points (pre, post, two-month follow up). Mediators, (i.e., active ingredients of interventions, e.g., self-compassion) and moderators (i.e., factors that change the strength of effects, e.g., implementation quality) will also be explored to understand how and for whom the intervention works best.

In addition to examining effectiveness, best practice guidelines in school-based research increasingly emphasise the importance of conducting implementation evaluations to assess how well programmes are delivered in schools and if programmes are delivered as intended [49, 51, 52]. Implementation evaluations are essential for providing insights into factors that may lead to a programme’s success or failure [51]. They can also inform efforts to guide sustainable delivery of programmes in schools. Thus, a mixed methods evaluation of implementation quality will be conducted to understand ‘why’ programme outcomes were observed and to inform efforts to optimise programme delivery in schools in the future. Aligning with previous studies [52, 53], the quality of implementation of BodyKind in schools will be assessed across four key implementation dimensions: (i) dosage, (ii) adherence, (iii) quality of delivery, and (iv) participant responsiveness.

This study will be the first to implement a cRCT design and implementation evaluation to examine BodyKind – an innovative, gender-inclusive, culturally sensitive programme designed to improve adolescents’ body image and psychological wellbeing. This research is anticipated to provide novel contributions to school-based body image intervention work.

We hypothesise that 1.) Compared to participants in the waitlist control, participants who receive the BodyKind programme will experience statistically significant increases in a.) body appreciation, psychological wellbeing, self-compassion and compassion for others and other’s bodies, social justice motivations and b.) significant decreases in body dissatisfaction and body ideal internalisation from pre- to post-intervention. 2.) Changes will be maintained in intervention groups at a 2-month follow up. 3.) Changes in body appreciation and body dissatisfaction will be mediated by self-compassion, compassion for others and body ideal internalisation. We make no a-priori assumptions about the directionality of mediation effects. 4.) Changes in body appreciation, body dissatisfaction and psychological wellbeing will be moderated by appearance-related social media use, appearance teasing, gender, baseline body appreciation, body dissatisfaction, psychological wellbeing and implementation quality. We make no a-priori assumptions about the directionality of moderation effects.

A mixed methods evaluation (non-directional) will be conducted to determine schools’ quality of implementation of BodyKind across key implementation dimensions. We expect that the programme 5.) will be considered acceptable and feasible by adolescents and teachers , 6.) can be implemented with high fidelity by teachers and that 7.) implementation quality may vary across schools and this may impact intervention effectiveness.

## Method

### Design

**Fig. 1:**
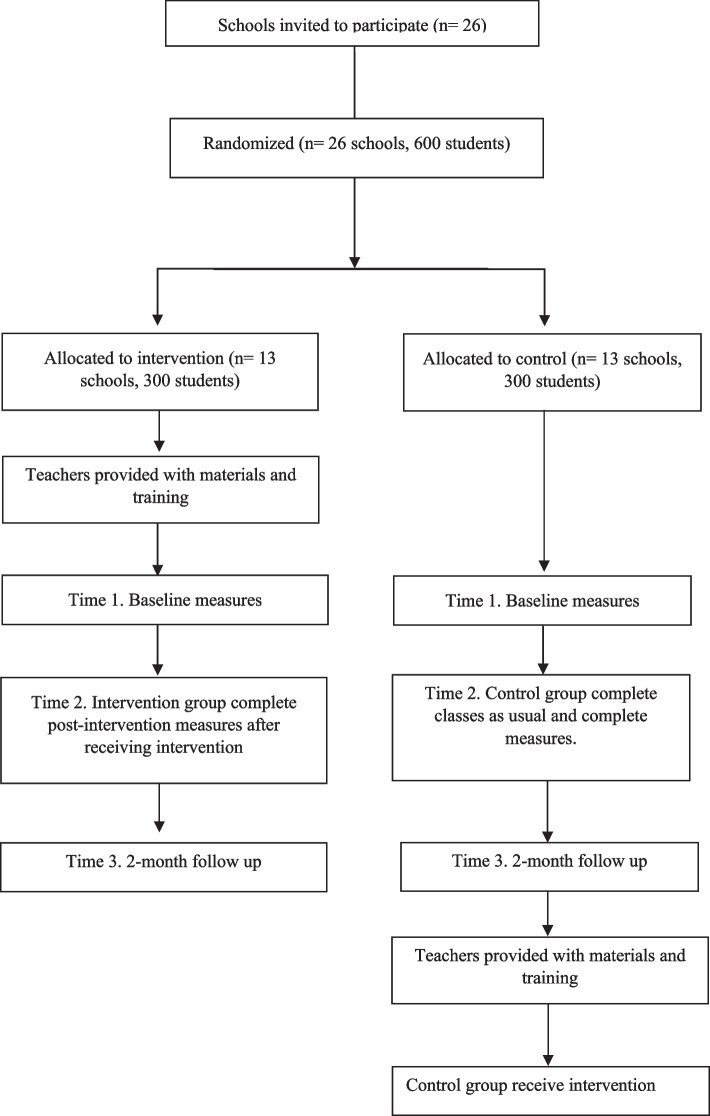
CONSORT recruitment diagram

This study will use a parallel two-arm cluster randomised control trial (cRCT) with equal allocation of schools to intervention or waitlist control conditions (see Fig. 1). Teachers in schools assigned to the intervention condition will receive training and will deliver the BodyKind programme to students, while the waitlist control will receive classes as usual. This will be a pragmatic trial, conducted under ‘real world’ conditions i.e., in a school setting, with teacher-led delivery during class time over a four-week period (1 lesson each week). Outcomes will be measured at three time points (pre-, post- and two-month follow up). As per [54], a two-month follow up was selected to fit within the academic school year. Waitlist control schools will complete teacher training and the BodyKind programme after the completion of measures. This study received ethics approval from University College Dublin University (HS-22-71) and was retrospectively registered on 10/10/2023 on ClinicalTrials.gov, NCT06076993.

### Participants

We aim to recruit a total of 600 students aged 15-17 years in Senior Cycle: Transition Year (a non-academically structured year in Irish secondary schools designed to facilitate the pursuit of alternative interests and self-discovery). An RMASS power analysis [55] based on parameters; small Cohen’s d effect size of .2, power .80, alpha .05, indicates that 504 students are required. A small effect size was selected in line with a meta-analysis of schools-based interventions for body image outcomes [14] which observed small effect sizes (d=.22 - .48) which despite being described as ‘small’ can have important real-world impact, particularly when scaled up across a whole school population [56]. To account for natural attrition and unanticipated events that reduce participant numbers (e.g., student absence due to illness) in cRCTs, we will recruit approximately 20% (*n=*100) more participants giving a total *N=*600. Given the variability in class size in Transition Year range in consent rates for active parental consent [57], and to guard against drop out at a cluster level we will recruit a minimum of 12 schools and a maximum of 26 schools with 1-2 fully qualified second-level teachers per school (range 12-52 teachers). Schools will be eligible if they are a second-level school in the Republic of Ireland that offers the Transition (TY) programme to students. Only data from full-time TY students aged 15-17 years (i.e., not exchange or short-term visiting students) will be included in analyses.

### Recruitment and randomisation

We will recruit students and teachers at the school level using 1.) Personal introductions to schools via our research partner, Educator School Centres Ireland’s (ESCI), who host continuing professional development (CPD) for teachers/schools on behalf of the Department of Education and Skills, 2.) Body Confident Schools webinar event for schools (a single online event for schools, hosted by ESCI & Jigsaw, The National Centre for Youth Mental Health), 3.) Advertisement on social media & website pages, 4.) Cold call invitations.

Schools will be selected on the basis of 1.) their expressed interest and 2.) their fit with recruitment criteria. Prior to randomisation, schools will be stratified based on school categorisation (e.g., urban/rural and single sex/coeducational) to ensure the gender and geographical location of participants in each treatment group is closely balanced. A stratified shortlist of 26 schools will be allocated using covariate-constrained randomisation, a type of minimisation randomisation, with the R statistical package cvcrand (https://cran.r-project.org/web/packages/cvcrand/cvcrand.pdf). Allocation will be constrained by a.) the number of boys and b.) the number of girls in TY in each school, and c.) the school’s location categorisation (urban/rural). Shortlisted schools registering interest that decline to participate after initial contact will be replaced by schools on the waitlist based on minimisation, or replacement by same category method. Given the nature of the study, blinding participants to their condition will not be possible.

### Procedure

Informed active consent will be sought from school principals (during recruitment) and we will also seek informed consent from teachers (before teacher training), parents (before baseline assessment) and assent from students (at baseline assessments). On receiving principal consent to participate, the researcher will liaise with a nominated staff member to coordinate involvement. Optional virtual ‘zoom check in’ meetings with the primary researcher will be offered to parents and students prior to participation in addition to teachers throughout the trial to address queries/issues about the programme/research. Students without consent to participate will be given the option to attend supervised study period or another school-based activity during time allotted to the intervention. Participants can withdraw from participation at any point without explanation.

Data will be collected via participant self-report to standardized questionnaires delivered in an online format using an online platform (e.g., SurveyMonkey) or in pen and paper format (as per the preferences of the school). Questionnaires will be administered to students by a member of the research team under exam-like conditions. In the post-intervention questionnaire, students in the experimental group will be asked additional questions about their perceptions of the programme. Teachers in the experimental group will also complete fidelity checklists after each lesson and a short questionnaire on completion about perceptions of the programme. On study completion, participants will receive a full debrief and contact details for relevant support services. Schools will receive a €200 financial honorarium for participating.

#### Teacher training

Teachers in the intervention group will attend a comprehensive 2.5 hour in person/online training session, delivered by the primary researcher (curriculum writer on the BodyKind programme and body image researcher) prior to programme delivery (August/September 2023). Teachers will be provided instruction on how to deliver the programme content and materials and encouraged to implement the programme as close to the teacher manual as possible over a four-week period. Teachers will receive a teacher training manual and access to over 2.5 hours of self-guided videos and online resources linked with the Be Real foundation to support delivery. Teachers in the waitlist condition will receive teacher training after data collection is completed.

### Measures

#### Student questionnaire

We will consult with student members of the Irish Second-Level Students Union of Ireland (ISSU), the national umbrella body for second-level students in Ireland, on the comprehension and suitability of the questionnaire schedule prior to administration to students. Two attention check items will be placed throughout the questionnaire to screen out participants who are not focused or paying attention (e.g., show you are paying attention by disagreeing below). Items will be randomized within scales to avoid order bias.

##### Demographic characteristics

Participants will report on age, gender and ethnicity. Single items assessed gender/sexual orientation, ethnicity/race, perceived body size, frequency of negative appearance commentary from peers based on appearance, and frequency of appearance-related social media use (i.e., hours spent using Instagram, TikTok, Snapchat or YouTube per day).

##### Primary outcomes

Body Appreciation Scale-2 (BAS-2; [58] is a 10-item scale. Responses to items such “I feel good in my body” are indicated on a 5-point Likert Scale (1-5), with higher scores indicating higher levels of body appreciation. BAS-2 demonstrates good validity and reliability among international adolescent samples [59].

Body Satisfaction Visual Analogue Scales (VAS; [60]) Items from the appearance/weight subscales of the Eating Disorder Examination Questionnaire will be adapted (EDEQ; [61]) as 100-point visual analogue scales (VAS) to assess state body satisfaction with various aspects of appearance. As per Durkin & Paxton [60] participants will use a 100-point slider 0 (not at all satisfied) and 100 (very satisfied) to rate how satisfied they feel with their body shape, weight, and size. Participants will also rate their satisfaction with height, muscle mass/tone and overall appearance. A mean score from the six appearance dimensions will be calculated, with higher scores representing higher state body satisfaction. This approach has been shown to have good convergent validity with the Eating Disorder Inventory Body Dissatisfaction Subscale in adolescents (*r* = -62; [60]).

The Five-Item World Health Organisation Wellbeing Index (WHO-5; [62]) is a unidimensional scale that measures emotional wellbeing using five positively worded items. Participants indicate the extent to which positive feelings, such as “I have felt calm and relaxed” were experienced over the last two weeks, using 6-point Likert scales ranging from 0 (not present) to 5 (constantly present). Raw scores are transformed to a score from 0 (worst thinkable well-being) to 100 (best thinkable well-being) with scores <50 suggesting poor emotional well-being [63]. The WHO-5 has been validated for use with adolescents and has adequate validity as an outcome measure in clinical trials [62, 63].

##### Secondary outcomes

The Self-Compassion Scale for Youth (SCS-Y;[64]) is a shortened and modified version of the Self-Compassion Scale (SCS; [65]) developed for use with adolescent populations. The SCS-Y is a 14-item scale that contains subscales for the aspects of compassion as identified by Neff [65]; self-kindness, self-judgement, common humanity, isolation, mindfulness and over identification. Participants respond to items such as “When I feel frustrated or disappointed, I think about it over and over again.” on 5-point Likert scales ranging from 1 (never) to 5 (always). Negatively worded items are reverse coded. Higher scores indicate higher levels of self-compassion. The SCS-Y has been validated among adolescents aged 12-17 years, where mean scores range between 1.0-2.49 (low), 2.5-3.5 (moderate), and 3.51-5.0 (high) and demonstrated good levels of internal consistency with Cronbach's alpha of >=.82 for each subscale.

Internalization-General subscale of the Sociocultural Attitudes Towards Appearance Scale-3 (SATAQ-3; [66]), will be used to assess internalization of social media ideals. Items were adapted to social media by substituting the words “social media” into item stems e.g. “I would like my body to look like people on social media”. The five-item scale is measured using five-point Likert scales ranging from 1 (definitely disagree) and 5 (definitely agree). Negatively worded items are reversed coded and items are averaged to yield a mean score, with higher scores indicating greater body ideal internalization. The original scale has been shown to be a reliable in adolescent girls and boys [67] and the adapted scale has been found demonstrate good psychometric properties and good levels of internal consistency, ranging from α = 0.75 among male, and α = 0.84 among female adolescents [43, 68].

The Appearance-Related Social Media Consciousness Scale (ASMC, [69]) is a 13-item scale that captures the extent to which individuals’ thoughts and behaviours reflect ongoing awareness of whether they might look attractive to a social media audience. Given that appearance-related use is a more important predictor of body dissatisfaction compared with general/non-visual social media use [70], this measure is important to include. Items such as “I think about how specific parts of my body will look when people see my pictures on social media” are measured using 7-point Likert scales ranging from 1 (Never) to 7 (Always). ASMC scores demonstrate strong internal consistency, convergent and incremental validity, and test-retest reliability in adolescent boys and girls [69].

Body Appreciation for Others Subscale of the Positive Body Image in Adolescents Scale (PBIAS; [71]) measures young people’s capacity to appreciate and respect other people’s diverse bodies and appearance, which is another element of positive body image. Participants respond on 7-point Likert scales ranging from 1 (strongly disagree) to 7 (strongly agree) to items such as “It is my hope that everyone is able to love their bodies as they are”. The body-other appreciation subscale shows good validity and, with McDonald’s (1999) coefficient omegas of ωh=.88 indicating good reliability.

The Compassion Towards Others Action Subscale of the Compassionate Engagement and Action Scales Youth (CEAS-Y, [72]), measures compassion in adolescents. The 4-item action orientation competency subscale focuses specifically on actions aimed to prevent and alleviate distress/suffering in others, such as “When others are distressed or upset by things, I am kind and supportive to them”. Participants respond using 10-point Likert scale from 0 (never) to 10 (always) with higher scores indicating higher compassionate action orientations towards others. This subscale shows good internal consistency with alpha values across items ranging between α=.84-.88 in adolescent boys and girls [72].

Appearance related social advocacy [46] is a purpose-built scale measuring appearance-related social justice/advocacy motivations/intentions which align with key aims of the BodyKind programme. Participants indicate on 5-point Likert Scales whether they 1 (disagree) or 5 (agree) with the three items asking if 1.) they are aware of experiences of people who look different to them, 2.) know how to promote fairness/equality for individuals regardless of appearance and 3.) have intentions to take action to challenge appearance bias. The three items are summed to give a total score, with higher scores indicating higher motivations/intentions for appearance-based social advocacy. This scale has demonstrated reasonable reliability in pilot studies with adolescents in the USA (α=.75) [46].

#### Student feedback questionnaire

After receiving the BodyKind programme, students in the intervention group will complete additional questions assessing perceptions of programme acceptability and perceived attainment of learning objectives. Open-ended questions will capture ways students have applied content to their personal lives, key programme take home messages and suggestions for programme improvement. See Additional file 1: Appendix A.

#### Teacher fidelity checklists

Teachers will complete a checklist at the end of each lesson indicating the extent to which they covered key aspects of the lessons. See Additional file 1: Appendix B.

#### Teacher questionnaire

After delivering the BodyKind programme, teachers will complete a questionnaire on their perceptions of their own delivery, student engagement, and perceived suitability of the programme for students and teacher’s attitudes towards the programme. Teachers will also be asked about their perceptions of the teacher training and teacher resources (manual/worksheets). Open ended questions will capture liked/disliked aspects of the programmes, teacher modifications to the programme and teacher suggestions for improving the programme to inform optimisation of programme implementation going forward. See Additional file 1: Appendix C.

### Implementation quality evaluation

Four dimensions of implementation quality will be assessed; 1. dosage (number of sessions delivered/attended), 2. fidelity (extent to which programme is delivered as intended), 3., quality (how well the programme is delivered by the facilitator), and 4., responsiveness (how well participants engage with the programme). As per Dowling & Barry [52], dimension scores will be combined to yield a total implementation quality index score which indicated schools as either high- or low- implementors. Dimensions will not be weighted as each dimension is considered equally important in this implementation quality index. Qualitative data will be used to expand on potential factors influencing programme implementation.

#### Dosage

Will be assessed via two indicators. 1.) Teachers will indicate if they delivered the lesson (Yes=1, No=0) and this will be summed across all four lessons, 2.) Students will indicate on the Student Feedback Questionnaire (Yes=1, No=0) if they remembered attending each of the four lessons, this will be summed across all lessons and student scores will be averaged for each school.

#### Fidelity

Although observational methods (i.e., audio recordings) are considered gold standard for assessing fidelity, they are time and resource intensive and may change behaviours of those being observed. Self-report checklists are widely used as practical and acceptable measures to assess fidelity [73, 74].

Fidelity, will be measured via two indicators. 1.) Teachers will indicate (1=yes, 0=no) whether they covered the key items/concepts on fidelity checklists for each lesson. There are 7 items on the checklist for each lesson, which will be summed across all lessons. 2.) In post-intervention questionnaires, teachers will be asked to indicate the degree to which they implemented the programme as instructed in the teacher training manual using visual analogue scales ranging from (0=Not at all, 100 = Very Much So).

#### Quality of delivery

Will be assessed using two indicators. 1. ) Students will indicate the extent to which they agree with the statement that the “Lessons were taught well by the teacher” using VAS scales ranging (0 = Disagree , 100 = Agree). 2.) Teachers will rate the extent to which they felt confident delivering the programme using VAS scales ranging (0=Disagree , 100=agree).

#### Responsiveness

Will be assessed using four indicators. 1.) Students’ responses to 6 items, indicating the extent to which they understood, paid attention, enjoyed, felt comfortable participating in the programme and believed the programme was relevant and important using VAS scales ranging from (0=Disagree , 100=agree). A mean score for each of the six items will be calculated for each student, and then, a mean score will be calculated by averaging the student scores within each school. 2.) Students’ overall rating of the programme from 0=very poor to 10=excellent.

3.) Teacher perceptions of student responsiveness will be assessed via 5 items, where teachers indicate the extent to which they believe students understood, engaged with and enjoyed the programme and the extent to which students gave their best effort in assessments using VAS scales ranging (0=Disagree , 100=Agree). A mean score for each of the five items will be calculated for each teacher. 4. Teachers also indicated their likelihood to recommend, implement the programme in the future (0=Disagree , 100=Agree) and their own satisfaction with the programme (0=Completely Dissatisfied, 100 = Completely Satisfied).

### Intervention

#### Programme development

BodyKind was developed in partnership with the Be Real USA, NFP (https://berealusa.org/) and an international team of body image researchers and health curricula writers. BodyKind content was based on the literature and existing programmes including The Body Project [19, 75] and Dove Confident Me [76], as well as pilot work from the self-compassion intervention Digital SMART [32]. BodyKind was refined through iterative trials with Be Real research interns as well as acceptability trials with students and health teachers in high schools across the USA [46].

#### Theoretical basis

BodyKind is designed to target risk factors for body dissatisfaction and strengthen positive body image using a multipronged approach. The programme incorporates cognitive dissonance, where students are encouraged to think critically about the costs (time, money, energy) of pursuing appearance ideals, with the view to reducing internalisation of and comparisons with body ideals [45, 75]. This programme also extends critical thinking to challenge unhelpful diet culture myths that underpin appearance concerns [77, 78]. Through a Myth Busting Activity, students learn to challenge the notion that “we can all achieve body ideals with sufficient hard work and effort” by learning about the biological limits of body change and how factors outside of our control, including genetics, influence shape/size, not only willpower and self-control [79]. Students also learn to challenge the ideas that “highlighting someone’s body ‘flaws’ will motivate them to engage in health behaviours” through learning about the counterproductive effects of body shaming for health [80]. Students also are encouraged to identify the issues with conflating health and appearance (i.e., recognising that appearance is often a poor indicator of physical/psychological health). It is anticipated that by challenging diet culture myths, there will an increased sense of awareness and understanding around complex factors that influence our bodies and body image and reductions in body-related self-blame and shame [29].

Students are also introduced to the concept of self-compassion which involves 1.) kindness, which involves non-judgement, self-care and empathy towards oneself, 2.) mindfulness, the capacity to view situations from a balanced perspective and 3.) common humanity, the ability to recognise that all humans are imperfect and experience similar difficulties in life [81]. Students reflect on how appearance pressures may show up in their own lives and on social media and learn, through experiential exercises, how to reframe unhelpful critical thoughts into kinder, soothing, self-compassionate ones in order to reduce body dissatisfaction. Learning to appreciate alternative aspects of themselves rather than overvaluing appearance and recognising that no-one is perfect (i.e., common humanity), is thought to improve functional and aesthetic body appreciation [27, 82].

This programme uniquely seeks to foster ‘compassion for others’ as a way of building positive body image [38]. Students learn about Appearance Bias -when a person is judged and treated differently based on how they look, rather than who they are or how they behave. Negative bias can lead to discrimination, and positive bias can manifest as "pretty privilege" where a person is treated better because they look like society's appearance ideals. Students read a collection of other people's body stories, gaining an awareness of how people of different sizes and abilities, with different skin shades, facial features, sexualities and gender representations experience the world. Students are also encouraged to think about ways they reduce appearance pressures and build a kinder environment for themselves and others, for example through reducing 'body talk' that reinforces unhelpful societal beliefs about appearance [37, 40].

Students explore how viewing their bodies as instruments, not ornaments; gratitude; and the acceptance of natural body diversity can lead to body confidence [83–85]. Students also hear about people's journeys from more negative to more positive body image, which is designed to help students model paths to more positive body image [86].

Incorporating social justice perspectives [35], students are given the tools to become agents for change and focus on real steps they can take to create a better world. Students receive guidance on creating their own Roadmap to Action which takes an idea from the issue stage to an action stage. They will learn tangible steps to create an action plan to address a body image issue they feel strongly about in this programme, empowering them to make positive changes in their communities and beyond which has been identified as a way to build positive body image in adolescents [84].

#### Structure & content

Each 50-minute lesson introduces a different theme/topic related to body image and incorporates a range of learning strategies to build skills in self-compassion, critical thinking, emotional regulation, social activism which are designed to support positive body image and psychological wellbeing. Table 1 outlines content and structure of the BodyKind programme.

**Table 1:** BodyKind lesson content overview

**Lesson**	**Learning objectives**	**Learning strategies**
1. Challenging appearance bias	• Understand Appearance Bias and the many ways people experience Appearance Pressure • Empathise with a diverse range of people and their experiences of their bodies & see own experience reflected in the stories • Learn how to incorporate Body Confidence tips into their lives (i.e., body functionality, gratitude, appreciation)	• Framing the topic of Appearance Bias • Body stories gallery walk to learn about other people’s experiences of body image. • Written reflection on ways to boost body confidence
2. Self-compassion and social media	• Students understand the impact of comparisons (on social media and elsewhere) • Students learn to recognize their own Inner Critic • Students learn to use Self-Compassion to be less harsh to themselves	• Self-compassion jigsaw activity to understand key components of self-compassion • Written reflection on self-compassionate social media use
3. Compassion for others	• Students challenge unhelpful cultural messages about appearance • Students recognize the impact of Body Talk with emphasis on the reaction of the person who is the subject of Body Talk • Students can identify ways to model other people’s journeys from negative to more positive body image.	• Myth busting activity on cultural messages • Discussion on teasing and compliments • Video of teenagers describing how they moved from a more negative to a more positive conceptualisation of their bodies • Letter to a friend activity where students apply concepts of self-compassion, body functionality, gratitude, body diversity, and Body Talk in addition to lessons in the stories of people who are now more body confident.
4. Taking action	• Determine an appearance-related topic they are interested in taking action on (e.g., challenging appearance bias) • Create a plan for positive change • Reflect on what they have learned in this process and this program	• Roadmap for taking an idea from issue to action guided activity • Bingo to revise key concepts.

## Data analysis

### Effectiveness trial

#### Data screening

Cases with more than 70% missing data, inappropriate responses, missed attention checks or no baseline data or surveys completed under 3 minutes or that cannot be matched with another time point, will be removed. This study will primarily report outcomes of Intention to Treat analyses, where all participants randomised to a condition are included in the linear mixed model. A per protocol analysis that comprises of those who provide outcome data and pass attention control checks will be reported as a secondary analysis.

Data will be screened for missing data. If the missing data is substantial (> 5%), a sensitivity analysis will be conducted. Multiple Imputation (MI) will be conducted using the Multiple Imputation Analysis function on SPSS. Under MI in SPSS, the Fully conditional specification (MCMC) method will be chosen. As recommended by Heymans and Eekhout [87], we will specify 50 iterations using Predictive Mean Matching (PMM) for continuous variables.

Prior to conducting inferential statistics, data will be screened for missingness, outliers, normality and model assumptions. Data will be summarised graphically and by using descriptive statistics.

#### Data analyses

Generalised linear mixed effects models (GLMM) will be used to determine change on outcome measures. Primary analysis will compare measures at time one (baseline) and two (post-intervention), to identify fixed effects of the intervention. Potential clustering effects will be examined and accounted for at the school level. The model will include 2 main effects (Repeated measures effect of Time and Between subjects’ effect of Condition) and one two-way interaction Time*Condition). Gender effects will also be considered. With gender in the analyses, the model will have three main effects and corresponding two-way and three-way interactions. Secondary analyses will examine data from time 3 (follow-up) to see if the intervention condition has maintained hypothesised changes in dependent variables. We will also assess the proportion of within-group and between-group variance by calculating the Intra Class Correlation (ICC). As complex models can fail to converge, if we encounter convergence issues in our models, we may consider dropping the random effect accounting for the smallest proportion of between-group variance.

Mediation analyses will investigate whether body ideal internalisation, self-compassion and compassion for others mediate change in outcomes of body dissatisfaction and positive body image from T1 to T2 in the intervention condition. Moderation analyses will investigate whether appearance-related social media use, appearance teasing, gender, school type, baseline body appreciation, body dissatisfaction, psychological wellbeing and implementation quality moderate changes from T1 to T2.

Exploratory analyses will investigate the extent to which results are sensitive to floor effects which are common in universal body image interventions. We will conduct a mixed-effects regression model that includes participants who report moderate levels of body dissatisfaction (i.e., boys and girls who score 1.5 standard deviations below mean for body dissatisfaction of their respective genders).

### Implementation quality evaluation

Implementation dimensions of: (i) dosage, (ii) adherence, (iii) quality of delivery, and (iv) responsiveness will be averaged to provide a total implementation quality score. Correlations will be used to explore relationships among the dimensions. Implementation quality will be explored as a moderator of intervention effectiveness.

As per [52], Total Implementation Quality = (Total Dosage + Total Adherence + Total Quality of Delivery + Total Participant Responsiveness) / 4.

#### Qualitative data analysis

Open ended responses to student and teacher questionnaires will be analysed using qualitative content analysis [88]. A coding frame will be established and used to code data. Two researchers will simultaneously code data and codes generated by both researchers will be compared and adjusted as necessary to reflect both analyses of the data. Cohen’s Kappa coefficient will be calculated to indicate levels of intercoder agreement.

## Discussion

Using a cluster RCT design, this study aims to evaluate the effectiveness of the BodyKind body image programme in improving adolescent body appreciation, psychological wellbeing and reducing body dissatisfaction (primary outcomes). In addition, this study will examine whether hypothesised changes in self-compassion, internalisation, compassion for others (secondary outcomes) mediate intervention effects. An implementation evaluation will assess implementation quality and potential moderators of intervention effects (e.g., gender, school type, implementation quality, baseline body image scores) will also be investigated.

### Strengths & limitations

Strengths of the proposed study include the rigorous cluster randomised control design, adherence to CONSORT guidelines [89] and 2-month follow up which will enable us to evaluate immediate and sustained intervention effects in a real-life school setting. This study also builds on pilot work both in the USA and Ireland [46] which evaluates a version of the programme culturally adapted for the Irish context and will contribute to the continuum of evidence in accordance with the British Medical Council’s Guidelines on the Design and Evaluation of Complex Interventions [47]. This study is also strengthened by the diverse range of schools it is seeking to recruit (i.e., diverse school type, size and geographical spread) which will improve generalisability of study findings. Additionally, the inclusion of coeducational (mixed) and single sex schools will enable us to assess if there are any differences in how the programme is received in mixed versus single sex groups, which is an important consideration for body image intervention programmes, and especially for this programme which is one of the first to adopt a more gender-inclusive approach [17]. In light of calls within the body image and public health fields to develop school-based interventions that are scalable and can be sustainably delivered via community providers [36, 45], the teacher-led delivery and inclusion of the mixed methods implementation evaluation are particular strengths of this study. Implementation evaluations are not routinely conducted in body image intervention work, and so by examining factors influencing implementation quality according to students and teachers, in addition to assessing mediators and moderators of this multicomponent body image programme, this study aims to provide important insights that can inform future body image intervention work.

Given the real-world context in which the trial will be conducted, there will be some limitations, such as the inability to blind participants or researchers to study allocation. Additionally, randomisation at a school-level rather than an individual level may increase the risk of selection bias and imperfect matching of intervention and control groups. Furthermore, there will be no active control group and data will be self-report and subject to bias. Due to time and resource constraints associated with such a large-scale project, longer follow up assessments, additional qualitative data such as parent reports, in-class observation of teacher delivery and interviews with teachers and students who received the programme will not be possible. Obtaining active parental consent is a common challenge in school-based research which can bias the sample and influence sample size [57, 90]. Other known challenges with school-based trials include student absenteeism, teacher fidelity to the intervention, attrition and missing data, which can impact data and study quality [50, 90].

### Implication for practice

BodyKind is one of the first body image intervention that blends complementary intervention approaches of self-compassion, compassion for others, cognitive dissonance, and social activism to address multiple facets of body image using a multipronged approach. It has been adapted to the Irish cultural context and aligns with wellbeing promotion framework in Ireland. BodyKind was developed to address an unmet need to provide an evidence-informed, gender inclusive, strengths-focused, universal body image interventions for older adolescents, and may provide important contributions to the field of body image intervention. BodyKind also represents a potentially useful, practical, resource for teachers to support student body image and wellbeing in the classroom.

### Supplementary Information

**Additional file 1:** **Appendix A.** Student Process Evaluation Questionnaire (Post-Intervention Questions; Experimental Group Only). **Appendix B.** Questionnaire schedule for teachers. **Appendix C.** Teacher Fidelity Checklist.

**Additional file 2.** SPIRIT 2013 Checklist: Recommended items to address in a clinical trial protocol and related documents*.

## Data Availability

Not applicable.
