# Supplementary material for: Protocol of a cluster randomised trial of BodyKind: a school-based body image programme for adolescents
Source: BMC Public Health. 2023 Nov 14;23:2246. doi: 10.1186/s12889-023-17002-x (PMC10647142; doi:10.1186/s12889-023-17002-x)
Supplement: Supplementary file 1 — Additional file 1: Appendix A. Student Process Evaluation Questionnaire (Post-Intervention Questions; Experimental Group Only). Appendix B. Questionnaire schedule for teachers. Appendix C. Teacher Fidelity Checklist. [file 12889_2023_17002_MOESM1_ESM.docx]

**Appendices**

**Appendix A**

**Student Process Evaluation Questionnaire (Post-Intervention Questions; Experimental Group Only)**

| **Item** | **Response** | **Author** |
| --- | --- | --- |
| Acceptability | | |
| 1. I enjoyed the lessons | 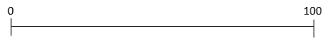Disagree Agree  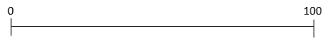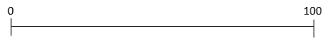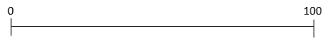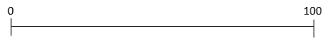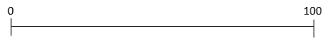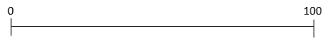  *(1 very poor - 10 excellent)* | [Qs 1-7: (Garbett et al., 2021)]  [Q8: Dowling & Barry, 2019] |
| 2. I understood what was being taught in the lessons |  |  |
| 3. I felt comfortable discussing the issues in a group with my classmates |  |  |
| 4. The lessons were taught well by the teacher |  |  |
| 5. It is important for young people to take part in lessons like these |  |  |
| 6. The lessons addressed issues that were relevant to young people |  |  |
| 7. I paid attention during lessons |  |  |
| 8. Overall I would rate the BodyKind programme as  . |  |  |
| Dosage /Attendance | | |
| Please indicate whether or not you remember attending the following lessons of the BodyKind programme  9. Lesson 1: Challenging appearance  10. Lesson 2: Self-compassion, comparisons & social media  11. Lesson 3: Compassion for others  12. Lesson 4. Becoming agents of change | Yes I did attend/No I did not attend | (Dowling & Barry, 2019) |
| Learning objectives | | |
| 13. Lesson 1: The programme helped me to notice and challenge the appearance biases that I encounter | 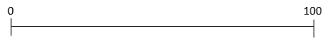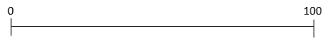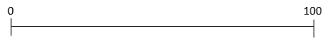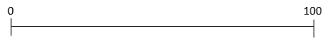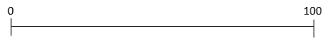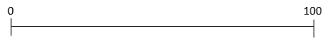Not at all to -very much | (BodyKind Pilot USA, 2023) |
| 14. Lesson 2: The programme helped me to use self-compassion to challenge the self-critical thoughts I feel. |  |  |
| 15. Lesson 3: The programme helped me to realise that being compassionate to others can help reduce appearance pressures that we all can feel. |  |  |
| 16. Lesson 4: I felt that the programme helped me learn how to tackle societal issues of concern to me |  |  |
| 17. This programme has helped me understand more about my own body image. |  |  |
| 18. This programme has helped me understand more about how other people might experience their bodies. |  |  |
| 19. This programme has given me tools/strategies to improve my body image |  |  |
| Take home/personal applications | | |
| 20. Please list one thing that you learned or that stood out for you from this curriculum | Open ended |  |
| 21. Please describe how you have applied one of the things you have learned in this program in your life. |  |  |
| 22. Do you have any suggestions to help us improve the programme |  |  |

**Appendix B**

**Questionnaire schedule for teachers**

| Question | Response  Not at all appropriate Very appropriate |
| --- | --- |
| Please indicate the degree to which the ‘BodyKind’ content was academically appropriate for your students | 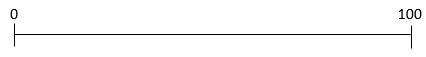 |
|  | Disagree…………………………………………………Agree |
| I felt confident delivering this programme | 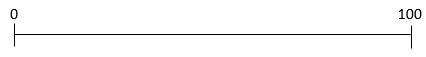 |
| There were no classroom management issues when delivering this programme | 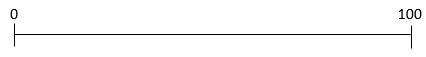 |

Teacher rating of student engagement with the program

| Question | Response  Disagree Agree |
| --- | --- |
| Students appeared to be actively engaged with the ‘BodyKind’ content during the lessons | 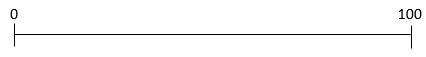 |
| Students appeared to enjoy the programme | 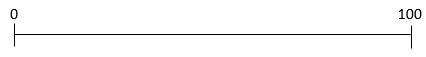 |
| Students appeared to understand the content of the curriculum | 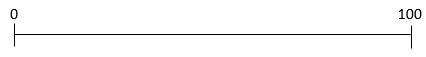 |
| Students appeared to give their best effort in the assessment component of this program. | 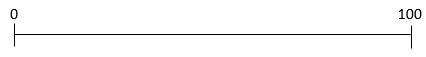 |
| Students appeared to complete the homework tasks | 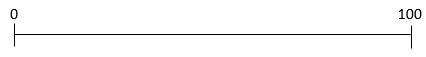 |

Teacher rating of the program

| Question | Response  Not at all Very much so |
| --- | --- |
| Please indicate the degree to which the BodyKind teacher manual provided you with all of the information that you needed to implement the programme | 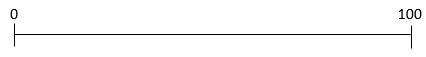 |
| Please indicate the degree to which the teacher education materials (i.e., training days/resources) prepared you to implement the programme | 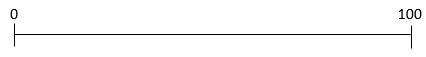 |
| Please indicate the degree to which delivered the programme as instructed in the teacher manual | 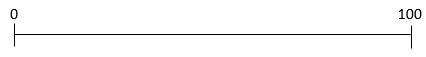 |
| Please outline the any ways in which you delivered the programme that were different to the teacher manual | Open ended response |
|  | Response  Not at all likely Extremely likely |
| How likely are you to recommend the ‘BodyKind’ program to other teachers | 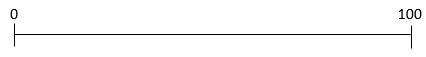 |
| How likely are you to implement the ‘BodyKind’ program in the future | 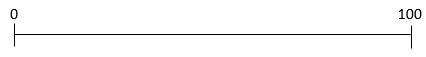 |
|  | Response  Completely dissatisfied Completely satisfied |
| Please rate your overall satisfaction with the ‘BodyKind’ program | 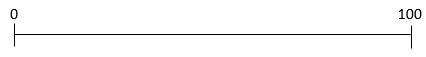 |

Teacher learnings from the programme

| The BodyKind programme made me question my body image beliefs and behaviours |  |
| --- | --- |
| The BodyKind program improved the way that I feel about my body. |  |

*Please respond to the open-ended questions below:

1. What were the best aspects of the BodyKind programme?
2. What did you like least about the BodyKind programme?
3. What would you change about the BodyKind programme?

Thank you for your responses to this survey

**Appendix C**

**Teacher Fidelity Checklist**

**Lesson 1 –Appearance Ideals and bias**

Please indicate by ticking which topics you covered in the session.

Date delivered: **_______________________**

| **Topic** | **Tick if completed** | **Comments** |
| --- | --- | --- |
| What is appearance bias? |  |  |
| Student Activity: Understanding Others Body Stories Gallery Walk |  |  |
| Reflection on Gallery Walk Activity |  |  |
| What are appearance pressures? |  |  |
| What are the costs of appearance pressures? |  |  |
| How can we build body confidence? |  |  |
| #Worksheet 1: Boosting Body Confidence |  |  |

**Lesson 2- Self-compassion and Social Media**

Please indicate by ticking which topics you covered in the session.

Date delivered: **_______________________**

| **Topic** | **Tick if completed** | **Comments** |
| --- | --- | --- |
| Student Activity: Self-Compassion Jigsaw |  |  |
| Do you compare yourself to others? What are the impact of comparisons? |  |  |
| What is the inner critic & how do we recognise it? |  |  |
| How do mindfulness and compassion help quieten the inner critic? |  |  |
| What is self-compassion & why is it helpful? |  |  |
| How can you speak more compassionately to yourself? |  |  |
| #Worksheet 2: Self-compassionate social media use |  |  |

**Lesson 3 – Compassion for others**

Please indicate by ticking which topics you covered in the session.

Date delivered: **_______________________**

| **Topic** | **Tick if completed** | **Comments** |
| --- | --- | --- |
| Student Activity: Myth busting |  |  |
| Myth 1: We can all have an ideal body with enough willpower and self-control. |  |  |
| Myth 2: Pointing out a person’s body “flaws” will motivate people to change them. |  |  |
| Myth 3: We need to look a certain way to be healthy |  |  |
| What is body-talk & what impact does it have? |  |  |
| Role Modelling Negative to Positive Body Image Journeys |  |  |
| #Worksheet 3: Letter to a friend |  |  |

**Lesson 4 – Taking action**

Please indicate by ticking which topics you covered in the session.

Date delivered: **_______________________**

| Topic | Tick if completed | Comments |
| --- | --- | --- |
| Student Activity: Roadmap for action |  |  |
| Selecting an issue: What issue resonated or impacted with you? |  |  |
| Select a plan of action: Public Awareness Campaign OR Create an artistic expression piece |  |  |
| 1.INVESTIGATE What others need to know about my topic to make change happen? |  |  |
| 2. ACTION PLAN: What steps do I need to take to make sure my plan is successful. |  |  |
| 3. EXECUTE How will I keep a record of my plan and know it is successful |  |  |
| 4. REFLECT: Project presentations & class reflection |  |  |
| #Worksheet 4: BodyKind Revision Bingo |  |  |
